# Generalized myoclonus following dobutamine stress echocardiography: a case report

**DOI:** 10.1093/ehjcr/ytag775

**Published:** 2026-10-21

**Authors:** Anastasios Bontozis, Alexandros Makris, Dimitrios Iliopoulos, Sotirios Kotoulas, Eftyhia Chamodraka

**Affiliations:** Department of Anesthesiology, Asklepieion General Hospital of Voula, Vas. Pavlou Ave. 1, Athens 16673, Greece; Department of Anesthesiology, Asklepieion General Hospital of Voula, Vas. Pavlou Ave. 1, Athens 16673, Greece; Department of Cardiology, Asklepieion General Hospital of Voula, Vas. Pavlou Ave. 1, Athens 16673, Greece; Department of Cardiology, Asklepieion General Hospital of Voula, Vas. Pavlou Ave. 1, Athens 16673, Greece; Department of Cardiology, Asklepieion General Hospital of Voula, Vas. Pavlou Ave. 1, Athens 16673, Greece

**Keywords:** Myoclonus, Dobutamine, Stress echocardiography, Adverse drug reaction, Neurological complication, Case report

## Abstract

**Background:**

Dobutamine stress echocardiography (DSE) is a widely used and generally safe non-invasive imaging modality for the assessment of myocardial ischaemia. Adverse effects are predominantly cardiovascular, whereas neurological complications are exceedingly rare. Dobutamine-induced myoclonus (DIM) has only rarely been reported, mainly in patients receiving prolonged dobutamine infusions for heart failure or cardiomyopathy.

**Case summary:**

A 47-year-old woman underwent DSE for evaluation of exertional chest pain. Following completion of a standard dobutamine protocol, she developed generalized rhythmic myoclonic jerks predominantly involving the axial musculature. The patient remained fully conscious and hemodynamically stable throughout the episode. Intravenous diazepam failed to improve symptoms, whereas complete resolution occurred after administration of intravenous valproic acid. Laboratory investigations, including electrolytes, renal function, and thyroid function tests, were normal. Electroencephalography and brain magnetic resonance imaging revealed no abnormalities. No recurrence was observed during hospitalization or follow-up.

**Discussion:**

To our knowledge, this is the first reported case of DIM occurring immediately after DSE. Unlike previously published cases, which predominantly involved patients with chronic kidney disease and developed after prolonged dobutamine exposure, our patient had normal renal function and developed symptoms immediately upon completion of the stress protocol. The pathophysiology remains uncertain, although increased central nervous system exposure to dobutamine has been proposed. Clinicians performing DSE should be aware of myoclonus as a rare but important neurological complication and should consider valproic acid when benzodiazepines fail to achieve symptom control.

Learning points

- Myoclonus is an extremely rare neurological complication of dobutamine, with fewer than 15 cases reported in the literature.
- Dobutamine-induced myoclonus can occur immediately after dobutamine stress echocardiography, not only during prolonged infusions for heart failure.
- Dobutamine-induced myoclonus may respond to valproic acid when benzodiazepines are ineffective.

## Introduction

Stress echocardiography (SE) is a well-established, non-invasive imaging modality widely used for the evaluation of suspected or known coronary artery disease.^1,2^ By assessing myocardial performance under physiological (exercise) or pharmacological stress, SE enables detection of inducible ischaemia and has evolved into an important diagnostic tool across a broad spectrum of cardiovascular conditions, including heart failure, cardiomyopathies and valvular disease.^3,4^

Dobutamine, a synthetic catecholamine, is the most commonly used agent for pharmacological SE. It primarily stimulates myocardial β1-adrenergic receptors, resulting in increased contractility and heart rate. Its action on β2-adrenergic receptors induces peripheral vasodilation, while a weaker α1-mediated vasoconstrictive effect is largely offset by its β2 activity.^5^ The overall haemodynamic effect is an increase in cardiac output and myocardial oxygen demand, facilitating the identification of flow-limiting coronary lesions.^6^

Dobutamine stress echocardiography (DSE) is generally safe, with a well-characterized adverse-effect profile, including arrhythmias, blood pressure fluctuations and angina. Non-specific symptoms such as headache, dyspnoea, nausea, palpitations, or anxiety may also transiently occur.^7^ Neurological adverse effects are exceedingly rare, and myoclonus following DSE has not previously been reported in the literature.^5^

We report the first case of generalized myoclonus occurring immediately after DSE, highlighting its distinguishing clinical features and management implications.

## Summary figure

**Figure:**
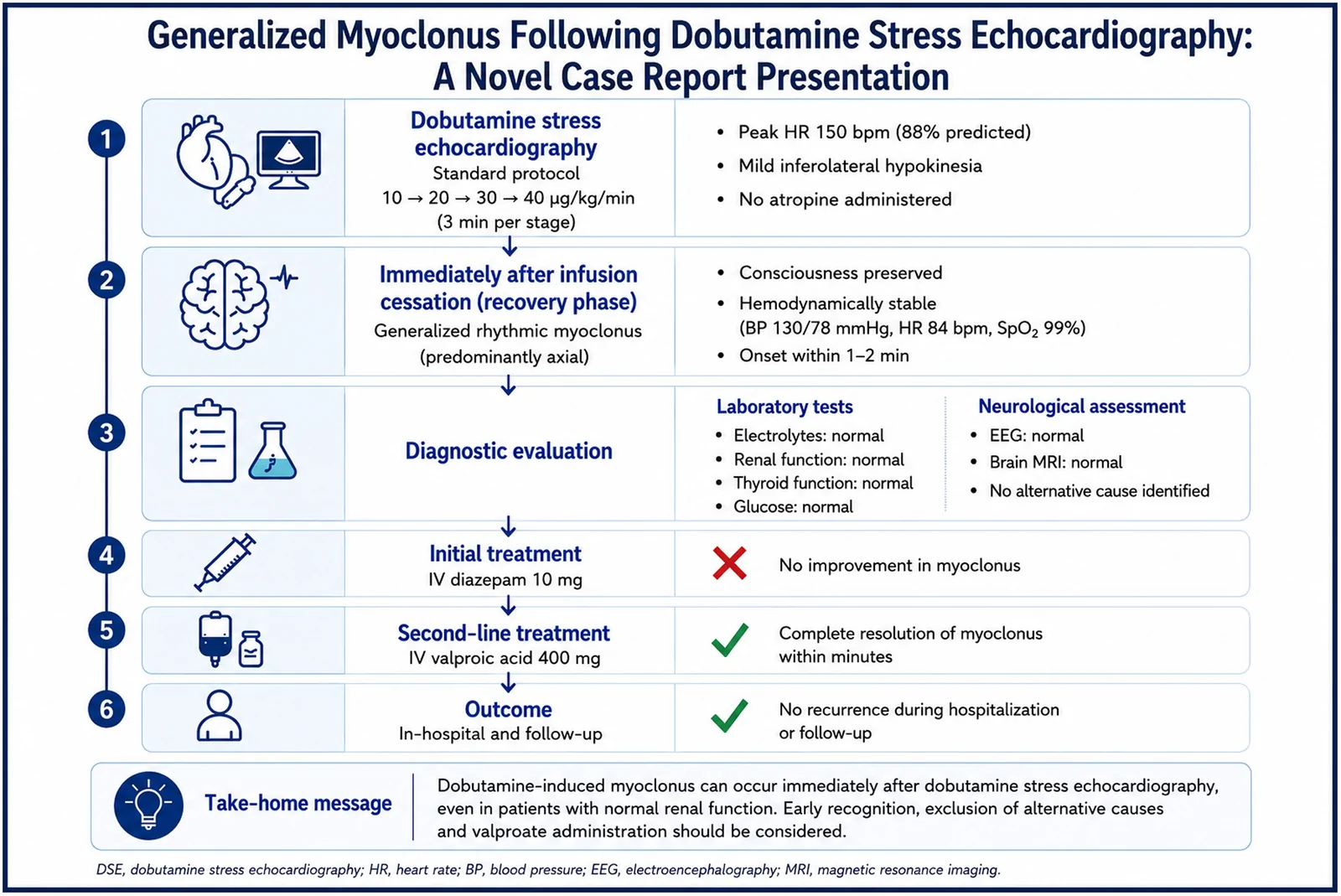


## Case presentation

A 47-year-old woman, with a body mass index of 29 kg/m^2^, was referred to the outpatient cardiology clinic for DSE due to recurrent episodes of exertional retrosternal chest discomfort suggestive of possible myocardial ischaemia. Her medical history was notable for arterial hypertension, dyslipidaemia, and hypothyroidism. Medications on admission included ramipril 2.5 mg and levothyroxine 62 μg once daily.

On initial clinical assessment, the patient was haemodynamically stable, with a heart rate of 72 beats per minute and blood pressure of 125/70 mmHg. Cardiovascular examination revealed a regular sinus rhythm without murmurs, and no peripheral oedema was noted. Lung auscultation was unremarkable. Baseline electrocardiography demonstrated normal sinus rhythm without ischaemic changes.

Transthoracic echocardiography at rest showed a normal-sized left ventricle with preserved systolic function (left ventricular ejection fraction 60%) and no regional wall motion abnormalities. No significant valvular pathology was identified.

DSE was performed using an Epiq CVx i 3D ultrasound system. Dobutamine was administered intravenously in a stepwise protocol, with each dose infused for 3 min before escalation, at incremental rates of 10, 20, 30, and 40 μg/kg/min. An intravenous ultrasound contrast agent was used throughout all stages of the study to optimize endocardial border delineation. During low-dose stages, there was an appropriate increase in global contractility without regional wall motion abnormalities. At peak stress, the patient achieved a maximum heart rate of 150 beats per minute and a peak blood pressure of 145/94 mmHg. She reported constrictive precordial chest pain at peak dose. Echocardiographic imaging at peak stress demonstrated an overall increase in systolic performance, with mild hypokinesia and reduced systolic thickening of the basal inferolateral wall. No electrocardiographic changes suggestive of ischaemia were observed during the test.

Immediately after the dobutamine infusion was discontinued, without gradual dose reduction, during the recovery phase, the patient developed generalized, rhythmic, symmetric myoclonic jerks predominantly involving the axial musculature, without facial involvement. There was no loss of consciousness, tongue biting, or urinary incontinence. The patient remained haemodynamically stable throughout.

Intravenous esmolol was administered, and the patient was subsequently admitted to the cardiology unit for further monitoring and management. The myoclonic episode lasted approximately one hour. Initial treatment with intravenous diazepam (10 mg) did not result in clinical improvement. Subsequently, intravenous valproic acid [1400 mg (20 mg/kg)] was administered, leading to complete resolution of the myoclonus.

Laboratory investigations revealed normal serum electrolytes, renal function and thyroid function tests. Cardiac troponin demonstrated a mild post-procedural elevation. A neurological evaluation was performed, and the episode was considered most likely drug-induced, related to dobutamine administration. Electroencephalography and brain magnetic resonance imaging did not reveal any pathological findings.

The patient remained clinically stable, and no recurrence of myoclonus was observed prior to discharge. At short-term follow-up, she remained asymptomatic, and no further neurological events were reported.

## Discussion

We describe the first reported case of dobutamine-induced myoclonus (DIM) occurring immediately after DSE. All previously published cases of DIM have been described during prolonged continuous dobutamine infusions administered for heart failure or ischaemic and non-ischaemic cardiomyopathies, with symptom onset ranging from 12 h to 3 weeks after initiation of treatment and resolution occurring between 30 min and 3 days after drug discontinuation.^5,8^ In contrast, our patient developed generalized myoclonus immediately after completion of the stress protocol, with symptoms persisting for approximately one hour, a markedly different temporal profile that suggests brief, high-dose catecholaminergic exposure may be sufficient to trigger this adverse effect in susceptible individuals.

A second distinguishing feature of our case is the absence of renal impairment. Published cases of DIM have predominantly involved patients with chronic kidney disease, often with end-stage renal disease.^5,8^ This has led to the hypothesis that impaired renal clearance of dobutamine or its metabolites contributes to central nervous system accumulation. Our patient had entirely normal renal function, indicating that chronic kidney disease is not a definitive prerequisite for the development of this complication, and that individual susceptibility factors such as blood-brain barrier permeability or central catecholaminergic sensitivity may play an important role.

The diagnosis of DIM was supported by the close temporal relationship between dobutamine administration and symptom onset, and by the systematic exclusion of alternative aetiologies. Electrolyte levels, renal and thyroid function tests were within normal limits, while electroencephalography and brain magnetic resonance imaging revealed no significant abnormalities. The patient remained fully conscious throughout the episode, making an epileptic event unlikely.

Management of DIM is not standardized because of its rarity. Previously reported cases have described the use of benzodiazepines, levetiracetam, and valproic acid, with variable clinical responses. In our patient, intravenous diazepam failed to improve symptoms, whereas complete resolution of myoclonus occurred shortly after administration of intravenous valproic acid. Although a causal relationship cannot be definitively established from a single case, valproic acid has recognized efficacy in the treatment of cortical and subcortical myoclonus. Its effects involve multiple mechanisms, including enhancement of GABAergic inhibition, reduction of glutamatergic excitation, and modulation of ionic conductance. Benzodiazepines primarily enhance GABAergic neurotransmission. However, the reason for the lack of response to diazepam in our patient remains uncertain, and no specific mechanism explaining differential responsiveness to these two agents in DIM has been established.^5,8–13^

The pathophysiology of DIM remains incompletely understood. The most widely proposed mechanism involves increased passage of dobutamine across the blood-brain barrier, possibly facilitated by altered P-glycoprotein efflux transporter activity, leading to central nervous system accumulation and neuronal hyperexcitability at dopaminergic and adrenergic receptors. A complementary hypothesis implicates impaired catabolism of dobutamine through catechol-*O*-methyltransferase inhibition, particularly in patients with advanced renal dysfunction. However, the latter mechanism appears less likely in our patient given the absence of renal impairment. Increased central nervous system exposure to dobutamine or heightened individual susceptibility to catecholaminergic stimulation may therefore represent more plausible explanations.^11^

Clinicians performing pharmacological SE should recognize DIM as a rare but important neurological complication. Prompt recognition, exclusion of alternative neurological causes, and appropriate symptomatic treatment may avoid unnecessary investigations and facilitate timely management.

## Lead author biography


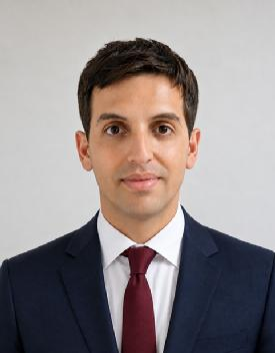


Anastasios Bontozis is an anaesthesiology resident at Asklepieion General Hospital of Voula in Athens, Greece. The author graduated from the National and Kapodistrian University of Athens School of Medicine and holds an MSc in Resuscitation from the same university. During the residency, the author completed clinical rotations in Cardiology and Cardiac Anaesthesia, which strengthened the author’s interest in perioperative cardiovascular medicine. The author’s clinical and research interests include cardiovascular anaesthesia, regional anaesthesia, perioperative medicine, and acute pain management. The author has presented research at national and international conferences and has contributed to publications in peer-reviewed medical journals.

## Data Availability

The data underlying this article will be shared on reasonable request to the corresponding author.
